# Management of Acute Type A Aortic Dissection at a Public Cardiac Center in the Northeast Region of Brazil

**DOI:** 10.21470/1678-9741-2020-0169

**Published:** 2021

**Authors:** Pablo Cesar Lustosa Barros Bezerra, Ricardo de Carvalho Lima, Pedro Rafael de Salerno, Antonio Cavalcanti de Albuquerque Martins, Geovanna Menezes de Medeiros Lustosa, Alvaro Monteiro Perazzo, Juliana Vieira de Oliveira Salerno, Carolina Vieira de Oliveira Salerno, Pedro Rafael Vieira de Oliveira Salerno

**Affiliations:** 1 Division of Cardiovascular Surgery, Pronto-Socorro Cardiológico de Pernambuco - PROCAPE, Universidade de Pernambuco - UPE, Recife, Pernambuco, Brazil.; 2 Postgraduation Department, Instituto de Medicina Integral Professor Fernando Figueira, Recife, Pernambuco, Brazil.; 3 Department of Cardiovascular Surgery, Universidade de Pernambuco - UPE, Recife, Pernambuco, Brazil.; 4 Department of Cardiovascular Surgery, Universidade de Pernambuco - UPE, Recife, Pernambuco, Brazil.; 5 Department of in General Surgery, Hospital da Restauração - SES-PE, Recife, Pernambuco, Brazil.; 6 Department of Universidade Federal de Pernambuco - UFPE, Recife, Pernambuco, Brazil.; 7 Department of Faculdade Pernambucana de Saúde - FPS, Recife, Pernambuco, Brazil.

**Keywords:** Aneurysm, Dissecting, Aorta, Hypertension, Aortic Diseases, Cardiovascular Diseases, Body Mass Index, Referral and Consultation

## Abstract

**Introduction:**

Aortic diseases are among the most serious cardiovascular diseases; the overall mortality rate due to diseases such as aneurysms and aortic dissections has been estimated at 2.78 per 100,000 persons in 2010, with a higher mortality rate in men than women. Our objective was to evaluate the epidemiological profile of patients with acute type A aortic dissection at a cardiology referral center.

**Methods:**

A retrospective cross-sectional study was performed at a public cardiac center with 24 patients hospitalized from 1/1/2016 to 12/31/2017 with a confirmed diagnosis of acute type A aortic dissection.

**Results:**

Twenty (83.3%) out of 24 patients underwent surgery and four (16.7%) did not undergo surgery. Among those who underwent surgery, 10 (50%) died and 10 (50%) were discharged, and all non-operated patients died (*P*=0.114) (Fisher's exact test). The male gender predominated (n=19, 79.2%), 86.7% (n=13) of the patients presented body mass index > 25 kg/m^2^, chest pain was found in 91.7% (n=22), and renal failure was present in 45.8% (n=11) of the cases. Hypertension predominated in 91.7% (n=22) and the main exam was aortic angiotomography in 79.2% (n=19) of the cases.

**Conclusion:**

The study presented a small sample size, making it impossible to associate the factors, although the service was considered a high-volume referral center. It is possible that the delay in arriving at the service and the accomplishment of invasive imaging with the use of contrast agents have aggravated the patients’ condition and have been decisive for the increase in lethality, which requires further studies.

**Table:** 

Abbreviations, acronyms & symbols				
AAS	= Acute aortic syndromes		MACCE	= Major adverse cardiovascular and cerebrovascular events
AD	= Aortic dissection		MRI	= Magnetic resonance imaging
BMI	= Body mass index		PAU	= Penetrating atherosclerotic ulcer
CT	= Computed tomography		PROCAPE	= Pronto-Socorro Cardiológico de Pernambuco
ICU	= Intensive care unit		SD	= Standard deviation
IMH	= Intramural haematoma		TEE	= Transesophageal echocardiogram
IRAD	= International Registry of Acute Aortic Dissection		TTE	= Transthoracic echocardiogram

## INTRODUCTION

Cardiovascular diseases are leading causes of death, accounting for 29.8% of all causes of death in Brazil in 2013^[1]^ and 31.3% of them in the world in 2015 (World Health Organization - WHO). Among all cardiovascular diseases, aortic diseases, divided into aneurysms, dissections, congenital diseases, and trauma, stand out^[2,3]^.

Acute aortic syndromes (AAS) are defined as emergency conditions with similar clinical characteristics involving the aorta. Physiopathologically, the origin is common to the various types of AAS that eventually start with rupture of the intima and middle layers of the aortic wall, which can result in intramural haematoma (IMH), penetrating atherosclerotic ulcer (PAU), or even in the separation of the layers of the aortic wall, causing aortic dissection (AD) or even complete aortic rupture^[3]^.

AAS occurs when a small lesion or ulcer allows blood to penetrate through the aortic lumen towards its middle layer or when the *vasa vasorum* ruptures, causing bleeding in the middle layer. The inflammatory response to blood in the middle layer can lead to aortic dilation and rupture^[3]^.

ADs are classified according to the time of symptom onset and type of symptom (according to the site of involvement). Regarding the time of symptom onset, they are usually classified as acute (up to 14 days), subacute (15-90 days), and chronic (> 90 days)^[3]^ Regarding the site of involvement, they are classified as Stanford^[4-9]^ type A (with involvement of the ascending aorta) and type B (without involvement of the ascending aorta)^[3-5]^.

ADs are believed to begin with the formation of a tear in the aortic intima that directly exposes an underlying diseased middle layer to the driving force of intraluminal blood, separating the layers of the aortic wall and the subsequent formation of a false lumen, with or without communication. In most cases, a small lesion of the intima is the starting condition, causing blood to travel through a dissection plane through the middle layer. This process causes both the rupture of the aorta, in the case of rupture of the adventitial layer, and the reentry into the true lumen through a second failure in the intimal layer^[3,6,7]^.

The dissection can be antegrade or retrograde. Complications include tamponade, aortic valve insufficiency, and proximal or distal malperfusion syndrome^[3,6,7]^ The inflammatory response to thrombosis of the middle layer is susceptible to initiate additional necrosis and apoptosis of smooth muscle cells, in addition to the degeneration of elastic tissue, factors that increase the risk of rupture of the middle layer^[3]^.

Currently, epidemiological data on AD are scarce. The incidence of AD is estimated at six cases for every 100,000 persons per year, with a higher mortality rate in men than in women and increasing with age^[3]^ The prognosis is worse in women, as a consequence of an atypical presentation of the disease and delayed diagnosis. The main risk factor associated with AD is hypertension, observed in 65-75% of the individuals, which is commonly poorly controlled^[3,6-8]^ In The International Registry of Acute Aortic Dissection (IRAD), the patients’ mean age was 63 years and 65% of them were men. Other associated risk factors are: pre-existing aortic disease or aortic valve disease, family history of aortic diseases, history of cardiac surgery, smoking, direct trauma to the chest, and use of intravenous drugs (*e.g*, cocaine and amphetamines)^[3,6,7]^.

Acute type A AD frequently presents with chest pain (in 80% of cases), with a sudden onset, but it can present as back pain or migratory pain. AD can complicate with aortic valve failure (40-75% of cases), cardiac tamponade (20% of cases), renal failure (20% of cases), myocardial ischemia or infarction (10-15%), and even coma or stroke (10% of cases)^[3,6,7]^.

The main objective of imaging studies in acute AD is the comprehensive assessment of the entire aorta, including its diameter, shape, and extent of dissection, involvement of the aortic valve, branches of the aorta, relationship with adjacent structures, and the presence of intramural thrombus^[3]^.

**Fig. 1A to C:**
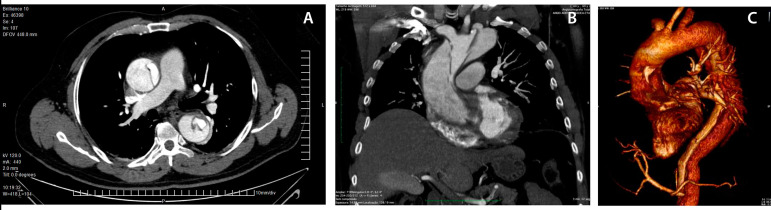
Angiotomography of one patient diagnosed with type A acute aortic dissection: A) transversal view; B) frontal view; C) 3D reconstruction view.

The main imaging tests used in the diagnosis of AD are transthoracic echocardiogram (TTE), transesophageal echocardiogram (TEE), computed tomography (CT), magnetic resonance imaging (MRI), and aortography. However, CT and MRI have been considered superior to TEE and TTE to assess the extension and involvement of branches in cases of AD, as well as for the diagnosis of IMH, PAU, and traumatic aortic injuries^[9]^ Figures 1A to 1C show three different views of the aorta from a CT angiotomography.

Drug therapy for pain control and hemodynamic control is essential. In acute type A AD, surgery is the treatment of choice^[3]^.

Acute type A AD has a 50% mortality rate in the first 48 hours if not operated^[3,5]^ Despite improvements in surgical and anesthetic techniques, perioperative mortality (25%) and neurological complications (18%) remain high^[3,9,10]^ However, surgery reduces mortality in one month from 90% to 30%^[3,7,9]^ The advantage of surgery over conservative treatment is particularly obvious in long-term follow-up^[5,9]^ Based on this evidence, all patients with acute type A AD should be referred to surgery; however, coma, shock secondary to pericardial tamponade, poor coronary or peripheral artery perfusion, and stroke are important predictive factors for postoperative mortality^[3,9,10]^.

As mentioned, acute type A AD is a disease with a high lethality rate, little is known about the epidemiology of type A AD in Brazil and in its North and Northeast regions, especially in the state of Pernambuco, as well as its impact on patient survival. Therefore, the aim of this study is to evaluate the epidemiology of patients with acute type A AD treated at the Pronto-Socorro Cardiológico de Pernambuco (PROCAPE) and to analyze the results obtained at the institution, so that it is possible to list proposals for improvements in local health care for this highly lethal condition.

## METHODS

### Study Design

The study was carried out at a public cardiac center in the Northeast of Brazil, being of a descriptive, retrospective cross-sectional nature, to assess the lethality and epidemiological profile of patients with acute type A AD, after confirmation of their diagnosis. Data collection was performed through electronic medical records, using as a sample the entire population of patients diagnosed with acute type A AD admitted to PROCAPE between 01/01/2016 and 12/31/2017.

Inclusion criteria were: patients diagnosed with AAS classified within ICD10-71 and who were hospitalized in that period. Exclusion criteria were: patients admitted and not diagnosed with acute type A AD during hospitalization.

The selected cases were included in a database spreadsheet previously coded in Microsoft Excel format with all the variables described by the researcher and contemplated by the patient in question, for further data analysis.

Two persons typed the data, at different times, making it possible to compare the two databases and identify possible typing errors, through consistency and cleaning tests. Only after completing these steps, the definitive database was used for statistical analysis.

The researcher analyzed the data using IBM Corp. Released 2011, IBM SPSS Statistics for Windows, Version 20, Armonk, NY: IBM Corp (or a newer version of it), with which were performed the calculation of the mortality rate of patients who underwent surgery and the calculation of the lethality rate of patients who did not undergo surgery. It was not possible to perform the logistic regression of the possible associated factors, as well as the calculation of average times and standard deviation between the procedures that patients went through during hospitalization, due to the low total number of cases found in this study, making it impossible to establish a relationship between outcomes and associated factors. No correlation analysis of any type of data was performed, nor any association between risk factors and death, due to the low total number of cases found in this study. The outcome tables were cross-checked with the surgery variable, and Fisher's exact test was used for analysis. In all stages of the analysis, the significance level of 5% was considered, adopting two-tailed *P*-values. Due to the factors mentioned above, multivariate analysis was dispensed with.

## Ethical Aspects

The study respected the ethical principles of the Declaration of Helsinki, resolution 466/2012, and was submitted to the Research Ethics Committee of the University of Pernambuco - UPE, under CAAE 88050618.2.0000.5192, approved on 06/14/2018. A waiver of informed consent was requested. Data confidentiality was guaranteed.

## RESULTS

In the period from 01/01/2016 to 12/31/2017, 100 patients with a diagnosis of aortic syndromes were admitted, of which 76 were excluded (62 for fulfilling the exclusion criteria and 14 for error in the electronic medical record) and 24 was the total of confirmed cases of acute type A AD.

Parametric variables (Table 1) did not differ between the groups, a significant relationship was found only between hypertension and surgical exposure, showing that among the cases that were operated on, 100% had hypertension, against 50% of those that were not operated on (*P*=0.022), although without clinical significance.

**Table 1:** Results of parametric variables (social, biological, clinical, surgical, and ICU).

Biological, social, clinical, and surgical variables	Total		Final outcome					Exposure				
	n	%	Death		Discharge		*P*-value*	Operated		Non-operated		*P*-value*
			n	%	n	%		n	%	n	%	
Gender							0.358					0.554
Male (n, %)	19	79.2	10	71.4	9	90.0		15	75.0	4	100.0	
Female (n, %)	5	20.8	4	28.6	1	10.0		5	25.0	0	0	
Proceedings							1.000					0.136
Metropolitan Region of Recife	18	75.0	11	78.6	7	70.0		16	80.0	2	50.0	
Other cities	4	16.7	2	14.3	2	20.0		2	10.0	2	50.0	
Other states	2	8.3	1	7.1	1	10.0		2	10.0	0	0	
Symptoms on admission^a^												
Chest pain	22	91.7	12	85.7	10	100.0	0.493	18	90.0	4	100.0	1.000
Dyspnea	4	16.7	4	28.6	-	-	0.114	2	10.0	2	50.0	0.115
Syncope	3	12.5	2	14.3	1	10.0	1.000	3	15.0	0	0	1.000
Dissection complications^b^												
Cardiac tamponade	2	8.3	2	14.3	-	-	0.493	1	5.0	1	25.0	0.312
Mesenteric ischemia	1	4.2	1	7.1	0	0.0	1.000	1	5.0	0	0.0	1.000
Major neurologic deficit (MACCE)	1	4.2	0	0.0	1	10.0	0.417	1	5.0	0	0.0	1.000
Limb ischemia	3	12.5	2	14.3	1	10.0	1.000	3	15.0	0	0.0	1.000
Renal failure	11	45.8	5	35.7	6	60.0	0.408	9	45.0	2	50.0	1.000
Myocardial ischemia or acute myocardial infarction	5	20.8	3	21.4	2	20.0	1.000	5	25.0	0	0.0	0.544
Comorbidities^c^												
Hypertension	22	91.7	12	85.7	10	100.0	0.493	20	100.0	2	50.0	0.022
Diabetes mellitus	3	13.0	1	7.1	2	20.0	0.550	3	15.0	0	0.0	1.000
Smoking	5	20.8	4	28.6	1	10.0	0.358	3	15.0	2	50.0	0.179
Medication in use												
Clopidogrel	2	8.3	1	7.1	1	10.0	1.000	2	10.0	0	0.0	1.000
Confirmatory diagnostic tests							0.059					1.000
Transthoracic echocardiogram	3	12.5	0	0.0	3	30.0		3	15.0	0	0.0	
Aortic angiotomography	19	79.2	13	92.9	6	60.0		15	75.0	4	100.0	
Aortography	2	8.3	1	7.1	1	10.0		2	10.0	0	0.0	
Diagnosis confirmed before admission	7	30.4	2	14.3	5	55.6	0.66	6	31.6	1	25.0	1.000
ICU variables												
ICU admission before surgery	16	80.0	11	78.6	7	70.0	0.665	16	80.0	2	50.0	0.251
Discharge from the ICU before surgery	2	10.0	1	7.1	1	10.0	1.000	2	10.0	0	0.0	1.000
Admission to the ICU after surgery			6	60.0	10	100.0	0.087					
Discharge from the ICU after surgery			0	0.0	10	100.0	0.000					
Non-operated patients admitted to the ICU	2	50.0										
Surgical variables												
Reoperation	4	20.0	2	20.0	2	20.0	1.000					
Number of type A AD surgeries in service per year (mean±SD)	10	±3										

^a,b,c^There may be more than one symptom, complication, and comorbidity in each case

* Fisher's exact test

AD=aortic dissection; ICU=intensive care unit; MACCE=major adverse cardiovascular and cerebrovascular events; SD=standard deviation

Non-parametric variables (Table 2) did not differ in most cases. An average body mass index (BMI) of 28.63 kg/m^2^ with a standard deviation of 3.00 kg/m^2^ among patients who died and an average BMI of 33.42 kg/m^2^ with a standard deviation of 5.6 kg/m^2^ among patients discharged were found (*P*=0.045). An average intensive care unit (ICU) length of stay after surgery of 3.32 days with a standard deviation of 4.45 days among patients who died and 5.75 days with a standard deviation of 3.13 days among patients who were discharged were observed (*P*=0.039). An average length of hospital stay of 5.81 days with a standard deviation of 7.56 days among patients who died and 26.97 days with a standard deviation of 20.45 days among those who were discharged from hospital were also found (*P*=0.001). A significant relationship was found only with the total length of hospital stay, with an average of 17.31 days and standard deviation of 18.14 days among patients who underwent surgery and 1.23 days and standard deviation of 1.22 days among those who did not undergo surgery (*P*=0.007).

**Table 2:** Results of non-parametric variables (age, BMI, and time).

Age, BMI, and time variables	Total			Final outcome					Exposure				
	n	Mean±SD	Median (Q1; Q3)	Death		Discharge		P-value*	Operated		Non-operated		P-value*
				n	Mean±SD	n	Mean±SD		n	Mean±SD	n	Mean±SD	
Age (years)	24	54.38±11.47	52.00 (48.25; 61.00)	14	56.71±11.20	10	51.10±11.59	0.427	20	54.80±11.25	4	52.25±14.12	0.392
BMI (kg/m^2^)	15	31.51±5.20	30.70 (28.81; 34.01)	6	28.63±3.00	9	33.42±5.60	0.045	14	31.49±5.40	1	31.75	0.817
Time from symptom onset to admission (in hours)	24	53.96±69.12	24.00 (7.00; 66.00)	14	57.64±79.54	10	48.80±54.97	0.882	20	58.85±74.77	4	29.50±16.27	0.784
Time between admission and completion of the confirmatory examination (in hours)	15	10.95±34.27	0.50 (0.38; 3.17)	11	13.08±40.10	4	5.11±7.75	0.557	12	13.20±38.29	3	1.98±2.69	0.885
Time of symptom onset and surgery (in days)	20	9.17±11.86	3.91 (1.45; 12.39)	10	8.17±8.79	10	10.17±14.75	0.880	-	-	-	-	-
Time between admission and surgery (in days)	20	6.72±11.07	1.58 (0.53; 8.65)	10	5.30±5.83	10	8.13±14.84	0.623	-	-	-	-	-
Time between symptom onset and death (days)	14	8.22±10.18	3.53 (2.17; 10.58)	-	-	-	-	-	10	10.52±11.34	4	2.46±1.35	0.496
Time between completion of the confirmatory examination and death (in days)	12	4.01±4.30	2.73 (0.69; 6.43)	12	4.01±4.30	-	-	-	9	5.06±4.47	3	0.87±1.40	0.052
Time between completion of the confirmatory exam and surgery (in days)	13	8.57±12.76	3.73 (0.94; 11.37)	9	3.85±4.65	4	19.20±19.40	0.064	13	8.57±12.76	-	-	-
Time between completion of the confirmatory examination and hospital discharge (in days)	4	41.00±26.64	37.69 (17.41; 67.91)	-	-	4	41.00±26.64	-	4	41.01±26.64	-	-	-
Time between surgery and death (in days)	6	3.69±4.43	2.08 (1.43; 5.33)	6	3.69±4.43	-	-	-	6	3.69±4.43	-	-	-
Time between surgery and discharge (in days)	10	18.84±12.80	15.14 (9.78; 23.98)	-	-	10	18.84±12.80	-	10	18.84±12.80	-	-	-
Length of ICU stay of non-operated patients who died (in days)	2	2.22±0.27	2.23 (2.03; 2.42)	2	2.22±0.27	-	-	-	-	-	2	2.22±0.27	-
Length of ICU stay before surgery (in days)	16	3.76±4.56	1.68 (0.38; 6.90)	9	4.34±4.91	7	3.02±4.34	0.223	16	3.76±4.56	-	-	-
Length of ICU stay after surgery (in days)	16	4.84±3.74	2.95 (1.99; 8.40)	6	3.32±4.45	10	5.75±3.13	0.039	16	4.84±3.74	-	-	-
Length of hospital stay of non-operated patients who died (in days)	4	1.23±1.22	1.18 (0.12; 2.39)	4	1.23±1.22	-	-	-	-	-	4	1.23±1.22	-
Length of hospital stay of operated patients who died (in days)	10	7.65±8.31	4.47 (1.99; 12.39)	10	7.65±8.31	-	-	-	10	7.65±8.31	-	-	-
Length of hospital stay of operated patients who were discharged from the hospital (in days)	10	26.97±20.45	20.48 (14.29; 33.05)	-	-	10	20.45±6.46	-	10	26.97±20.45	-	-	-
Total ICU time (in days)	22	6.46±4.59	5.19 (2.33; 10.24)	12	5.29±5.08	10	7.87±3.68	0.166	20	6.88±4.60	2	2.22±0.27	0.138
ICU time of non-operated patients	2	2.22±0.27	2.23 (2.03; 2.42)	2	2.22±0.27	-	-	-	-	-	2	2.22±0.27	-
Total length of stay (in days)	24	14.63±17.59	9.85 (2.38; 21.80)	14	5.81±7.56	10	26.97±20.45	0.001	20	17.31±18.14	4	1.23±1.22	0.007

Mann-Whitney's U test

BMI=body mass index; ICU=intensive care unit; SD=standard deviation

**Fig. 2:**
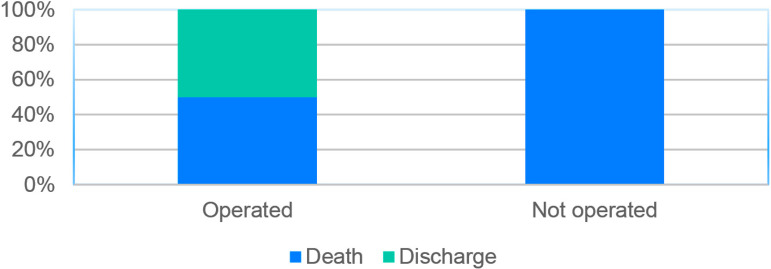
Crossover between surgical exposure and the final outcome (death or discharge). *Fisher’s exact test. P=0.114.

Between the final outcome and the surgical intervention, and as an exposure factor and main modifier of prognosis (Figura 2), it was shown that 100% of the patients who did not undergo surgery died during hospitalization and, of those who had surgery, 50% survived (*P*=0.114). Among these non-operated patients, two decided not to undergo surgery and two died before surgery.

In the present study, no patient was positive among the variables: ethnicity, pleural effusion, Marfan syndrome or its spectrum, peripheral arterial occlusive disease, previous stroke, use of ticagrelor, and use of oral anticoagulants. No patient underwent TEE or cardiac nuclear magnetic resonance as diagnostic test for AD or even as a complementary test during the investigation.

## DISCUSSION

The study obtained a small sample, despite capturing all cases of acute type A AD admitted to the service during the period, for significant analytical evaluation. Despite this low number of cases, the total number of surgeries performed per year at our service, an average of 10 cases per year, considered by many to be a high-volume referral center^[11]^, was similar to that of the Bristol Heart Institute, a referral hospital in cardiology in England, which published in 2017 an average of 12 cases operated per year, over a period of 17 years^[12]^.

The mean age found at 54.4 years is similar to that found in other studies, ranging from 43.5 to 61 years^[7,13]^ The male gender predominated with 79.2% of cases, ranging from 62.0 to 74.0% in the literature^[7,13]^ The average BMI was 31.5 kg/m^2^, similar to other studies^[7,13]^, but considering BMI ≥ 30 as suggestive of obesity and that 60.0% of the cases in this study were in this value range, the present study demonstrated a population with an obesity profile much higher than that evidenced in the literature, around 7.5%^[7,13]^, and this factor may have contributed to the increase in mortality. Among the symptoms, chest pain was found in 91.7% of cases and syncope in 12.5%, both similar to IRAD^[7,14]^.

Among the complications related to dissection, the high frequency of renal failure and myocardial ischemia stands out around 45.8% and 20.8% of cases, respectively, against 9.0-35.0% and 2.5-19.2% described in the literature, respectively^[7,13,14]^ Patients were more hypertensive (91.7% of cases) than those registered in other services, which ranged from 58.7 to 89.0% of cases^[7,13,14]^ The most performed exam was aortic angiotomography, in 79.2% of cases, against 50.2% of the cases reported in the IRAD^[7,14]^ There is also a greater number of aortographies in the diagnosis (8.3% of cases), higher than that of IRAD (about 2% of cases)^[7,14]^.

The average time from symptom onset to hospital admission was 53.96 hours (median=24 hours). This delay in hospital admission meant that patients were seen at a later stage^[11]^, which may have contributed for increased mortality, even with a response time between admission and surgery with a median of 1.58 days - less than 48 hours, as recommended, but still far above the ideal, which is 5-6 hours^[8,11,13]^ The total ICU length of stay and hospitalization times were similar to those found in other studies^[8,11,13]^ The frequencies of reoperations and intraoperative death, both of 20.0%, were higher than the average^[8,11,13]^, being more similar to the rates found among older patients, such as octogenarians^[15]^.

The lethality among the operated cases (50.0%) was higher than the lethality found in most studies, which varies between 21.0 and 30.0%^[7,14]^, but it is similar to studies carried out with patients over 75 years old, which varies between 42.0% and 83.0%^[15]^ In-hospital mortality among non-operated cases was 100.0%, much higher than the IRAD record^[7]^ It is possible that the delay in reaching a referral center has aggravated the condition of patients seen at the service, as well as the performance of more invasive imaging tests, with the use of contrast, which may also have contributed to the high rate of renal failure among patients, and the sum of these factors has been decisive for the increase in lethality; however, further studies are needed to prove or infer this increased risk.

Our service carries out the most diverse and recent surgical techniques and strategies (*e.g*, selective cerebral perfusion and endovascular treatment) well established in the treatment of acute type A AD, even though there is no significant difference between the outcomes with more aggressive strategies^[12,16-18]^, however, a rational strategy for the use of surgical techniques for the treatment of this condition could be implemented in the service^[12]^.

It is important that our institution, as well as all healthcare institutions, implements measures to improve the quality of the services provided, since the diagnosis of AD can be very laborious in non-specialized services^[20,21]^ To make our institution a center specialized in the treatment of aortic diseases to which all patients from the public health system with this condition should be referred, further increasing its surgical volume and improving the final results of the treatment^[11,20,21]^, it is crucial the implementation of local or even national registries, such as IRAD^[7]^ and Nordic Consortium for Acute Type A Aortic Dissection^[11]^; implementation of the institution's own protocols based on the literature, establishing a routine from the initial care at the institution to rational strategies in choosing the surgical technique, as well as using the data from this work, and always improving with the use of forthcoming researches carried out at the institution^[12,20,22]^; and implementation of a protocol for safe surgery and improvements in health care: definition of local objectives (emergency, ICUs, wards, operating room, and all sectors involved); establishment of measures; selection, testing, and implementation of changes; and, lastly, dissemination of changes to the entire hospital^[21]^ Rationality, always respecting the Hippocratic principle *primum non nocere*, knowing that the more you move away from evidence-based practices and the care to see, feel, and listen to the patient, the closer you get to iatrogeny, thus avoiding unnecessary procedures and conduct that can delay, hinder, or even prevent the success of adequate treatment, which can culminate in the patient's death^[23]^.

The future of the treatment of acute type A AD is closer, with the emergence and improvement of endovascular techniques, now well established for type B cases^[24]^ Although conventional open surgical treatment is still the gold standard^[3]^, some centers have already started treating these cases endovascularly, in situations where surgical risk is prohibitive^[24]^ It is expected that the development and improvement of these new techniques will reduce the cost of treating this condition, making it more widespread and accessible, in addition to bringing new perspectives for the treatment of acute type A AD^[17]^ The improvement and development of broader risk scores, such as the Society of Thoracic Surgeons score and the European System for Cardiac Operation Risk Evaluation score II^[14,25]^, to better contemplate the surgical risk of these patients, especially among the elderly (> 75 years), with the use other variables, such as the frailty scale, can determine a risk more compatible with reality^[15,25]^.

Limitations

This is a single-center, non-randomized retrospective study, with a more descriptive character and a small sample. Our service is relatively recent, in addition to having just become a referral center in the state for this condition. We have a multi-surgeon profile for aortic surgery.

## CONCLUSION

The sample size was small, although our institution is considered a high-volume referral center; however, despite being a low-incidence disease, the sample clearly demonstrated the compatibility of its size with the literature. Lethality is high, and an even greater lethality was found in this study, possibly due to the delay between the symptom onset and admission to the service due to delayed diagnosis and referral of primary and secondary services, further aggravating patients, as well as due to the performance of more invasive imaging exams, using contrast, may also have contributed to the high rate of renal failure among patients. The sum of these factors has been decisive for increasing the lethality, however, more studies are needed to prove or infer a cause-effect relationship, as well as to implement actions for the early identification of these patients in non-specialized services, to reduce the time taken to referral services in the treatment of acute type A AD.

**Table:** 

Authors' roles & responsibilities	
PCLBB	Substantial contributions to the conception or design of the work; or the acquisition, analysis, or interpretation of data for the work; drafting the work or revising it critically for important intellectual content; final approval of the version to be published
RCL	Drafting the work or revising it critically for important intellectual content; final approval of the version to be published
PRS	Drafting the work or revising it critically for important intellectual content; final approval of the version to be published
ACAM	Drafting the work or revising it critically for important intellectual content; final approval of the version to be published
GMML	Drafting the work or revising it critically for important intellectual content; final approval of the version to be published
AMP	Drafting the work or revising it critically for important intellectual content
JVOS	Substantial contributions to the acquisition, analysis, or interpretation of data for the work
CVOS	Substantial contributions to the acquisition, analysis, or interpretation of data for the work
PRVOS	Substantial contributions to the acquisition, analysis, or interpretation of data for the work
